# Development of a Strategic Tool for Shared Decision-Making in the Use of Antidepressants among Patients with Major Depressive Disorder: A Focus Group Study

**DOI:** 10.3390/ijerph15071402

**Published:** 2018-07-03

**Authors:** Syahrir Zaini, Harvin Anbu Manivanna Bharathy, Ahmad Hatim Sulaiman, Jesjeet Singh Gill, Koh Ong Hui, Hasniza Zaman Huri, Siti Hadijah Shamsudin, Ng Chong Guan

**Affiliations:** 1 Department of Psychological Medicine, Faculty of Medicine, University of Malaya, Kuala Lumpur 50603, Malaysia; anbuharvin94@gmail.com (H.A.M.B.); hatim@um.edu.my (A.H.S); jesjeet@um.edu.my (J.S.G.); ohkoh@um.edu.my (K.O.H.); chong_guan@um.edu.my (N.C.G.); 2 Department of Pharmacy Practice, Kulliyyah of Pharmacy, International Islamic University Malaysia, Kuantan 25200, Malaysia; shadijah@iium.edu.my; 3 Department of Pharmacy, Faculty of Medicine, University of Malaya, Kuala Lumpur 50603, Malaysia; hasnizazh@um.edu.my

**Keywords:** shared decision-making, antidepressants, depression, focus group discussion

## Abstract

Shared decision-making (SDM) has been recognized as an important tool in the mental health field and considered as a crucial component of patient-centered care. Therefore, the purpose of this study was to develop a strategic tool towards the promotion and implementation of SDM in the use of antidepressants among patients with major depressive disorder. Nineteen doctors and 11 major depressive disorder patients who are involved in psychiatric outpatient clinic appointments were purposively selected and recruited to participate in one of six focus groups in a large teaching hospital in Malaysia. Focus groups were transcribed verbatim and analyzed using a thematic approach to identify current views on providing information needed for SDM practice towards its implementation in near future. Patients’ and doctors’ views were organized into six major themes, which are; summary of treatment options, correct ways of taking medication, potential side effects of treatments related to patients, sharing of case study related to the treatment options, cost of treatment options, and input from pharmacist. The information may be included in the SDM tool which can be useful to inform further research efforts and developments that contribute towards the successful implementation of SDM into clinical practice.

## 1. Introduction

Shared decision-making (SDM) has been recognized as an important tool in the mental health field and considered as a crucial component of patient-centered care [1,2]. SDM has been known as an approach where clinicians and patients share the best available evidence when faced with the task of making decisions, and where patients’ autonomy and engagement are promoted so that they can consider options in achieving informed preferences [3]. Some countries provided mental health policy and clinical guidelines that facilitate service user participation in the mental health care planning and service delivery [4,5]. However, many service users including patients are still experiencing being excluded from their management plan [6].

In medical decision-making, participation of patient is influenced by autonomy, which refers to the decision-making dimension of the patient’s role and improving patient autonomy means assisting patients to decide on their own [7]. Over time, doctor–patient relationships have been practiced differently. At the beginning, the traditional “paternalistic” model consisted of only the clinician making decisions [8]. In the current situation, an “informed” decision making model has been developed, whereby information flows from the clinician but the patient is making decisions [9].

SDM has been adopted as the central to the recovery model and has led to an increasing emphasis of patients’ role as active participants in their treatment plan [10]. In terms of mental health care, inter-professional collaborative relationships and practices are important to encourage more active participation of patients and their caregivers in the process [11,12]. The successful collaboration in mental health care requires a free flow of information and feedback sharing among all participants so that each of them are on track with any changes that will occur throughout the treatment process [13].

Major depression is one of the common psychiatric disorders linked to diminished role functioning and quality of life, medical morbidity, and mortality [14,15]. Major depression has been ranked as the fourth leading cause of disability worldwide by the World Health Organization [16]. Depression is associated with work disabilities among long-term unemployed people [17], and the risk of developing depression is increased among those who anticipated job insecurity [18]. The average total cost of patients with depression is US$7638 per patient-year and indirect costs (e.g., unemployment and loss of productivity) dominated the total costs [19]. The high cost associated with depression is a tremendous burden for patients with depression and unemployment.

Depression has been predicted to be the second leading cause of disability by 2020 [20]. Therefore, appropriate management which includes treatment and prevention for depression should be given priority in the 21st century. The most commonly used medication for depression is antidepressant, and the prescribing trend has increased tremendously over the last decades [21,22,23,24].

On top of that, antidepressants have similar problem like other medications. The non-adherence prevalence of antidepressants is high. Almost half (56%) of the patients will discontinue their antidepressants within the first six months [25,26]. Several studies estimated that some (6–12%) patients never start the treatment at all [27,28]. The term used for this situation is known as initial medical non-adherence (IMNA) or primary non-adherence [29]. IMNA is associated with increased usage of health care services and decreased rate of productivity in chronic and acute diseases [30]. Non-adherence to antidepressants will increase treatment costs since it is associated with worse clinical outcomes and more health care resources need to be provided to manage the situation [31].

The reasons of IMNA may be due to fear of side effects, limited guidance by health care professionals during treatment, lack of knowledge, a negative perception towards antidepressant use and the depression itself [32]. It is suggested that doctors, pharmacists, and other health care professionals should be more supportive during the initiation phase of antidepressant treatment so that positive effects of SDM on adherence and depression outcomes can be obtained [33]. It has been proven that even patients with severe mental illness require full information related to their treatment and they are willing to be actively involved in this professional relationship [9,34,35,36].

SDM in mental health care involving doctors and patients have been practiced in other countries, with various decision support tools have been developed [37]. Among the challenges listed for the successful of SDM in mental health include integrating SDM with other recovery-supporting interventions, creating widespread access to high-quality decision support tools, and responding to cultural changes as patients develop the normal expectations of citizenship [38]. Therefore, actual data from Malaysia is important, since it is still currently lacking.

Until now, little has been known about major depressive disorder patients’ views on SDM. Even though SDM has been introduced in Malaysia since 2010, but there has been no widespread implementation of this concept [39]. In order to provide optimal guidance on SDM for antidepressant use in major depressive disorder patients, the experiences and views of both doctors and patients should be known and taken into consideration. Therefore, the objective of this study was to develop a strategic tool that is informed by doctors’ and patients’ perspectives towards the promotion and implementation of SDM in the use of antidepressants among patients with major depressive disorder. 

## 2. Materials and Methods

### Setting and Sample

This study took place in the Psychological Medicine Department, University Malaya Medical Centre, which is a teaching hospital in Malaysia. Focus group study was employed, that is one type of qualitative study design. Focus group was chosen to gain an in-depth and rich detail understanding of doctors’ and patients’ opinions on implementing SDM in the use of antidepressants among patients with major depressive disorder.

Doctors and patients were recruited through a purposeful sampling approach, which include the ‘snowball’ strategy. Purposeful sampling has been used widely for the identification and selection of information-rich cases in qualitative research. ‘Snowball’ is one of the strategies under purposeful sampling in implementation research [40]. The objective of this strategy is to identify the cases of interest from sampling people who know other people that are generally suitable to participate in this focus group study.

Specifically, this strategy started with the identification of potential participants from the professional contacts of the research team. A co-author (N.C.G.), who is currently working in this center, invited suitable doctors and patients to be included in the study. Then, this strategy continued with those invited doctors and patients invited their colleagues that might be similar in characteristics with them. Finally, a total number of 30 participants (19 doctors and 11 patients) were included in the study and analysis. Ethical approval for the study was obtained from the Medical Research Ethics Committee, University Malaya Medical Centre (MRECID.NO: 2017816-5498). 

All 19 doctors agreed to participate after screening of the suitable criteria by the research team. Eligible doctors had provided antidepressant prescribing within the previous six months in an outpatient and/or inpatient setting. Similarly, for the patients, after screening of the suitable criteria by the research team, all 11 patients that were identified, agreed to participate in this study. Eligible patients included those diagnosed with major depressive disorder, whether under current ward admission or follow-up outpatient clinics. They were high-functioning patients with depression, under follow up of doctors from the Department of Psychological Medicine, in a teaching hospital in the city. High-functioning depression refers to those who have low-grade depression marked by lagging energy or fatigue, which does not meet the criteria of more severe major depressive disorder [41].

Discussions were facilitated by a research team that acted as the focus group moderator. Additionally, another research team was also present in each session to be the note taker. Before starting any of the focus group sessions, the participants had the study explained to them in detail by the moderator. Interested participants completed a demographic profile form, provided consent, and attended focus group according to their time preferences. 

Several open-ended questions that were asked for all groups (patients and doctors) as adapted from previous study [42]: “How can patients and doctors negotiate what kind of treatment is chosen?”, “What can patients contribute so that we reach decisions that are reasonable for both patients and physicians?”, and “What else can patients do to contribute to successful treatment?”

These questions were included in the development of focus group topic guide (Supplementary File), as adapted from previous study [43]. This guide included a general question-by-question outline used by the moderator during each of the focus group session. Questions proceed from general and nonthreatening topics to more particular and potentially specific topics. This also gave the moderator chances to probe responses in obtaining specific opinions related to the topic of interest [44].

Audio recordings were done for all six focus groups with the participants’ permissions. Then the recordings were transcribed verbatim to facilitate thematic analysis. Several steps of data analysis were done, including data familiarization, initial code creation, recurrent pattern identification, theme development, and review. These steps were guided by La Pelle [45] article entitled “Simplifying Qualitative Data Analysis Using General Purpose Software Tools”. Qualitative data coding and retrieving were done using Microsoft Word macros [46]. Data saturation was reached with the third patient focus group and the third doctor focus group.

## 3. Results

Three focus group discussions (FGDs) with doctors and three FGDs with patients were conducted within the period of September until October 2017, at the Department of Psychological Medicine, University Malaya Medical Centre (UMMC). A total number of 30 participants (19 doctors and 11 patients) were included in the study and analysis. The size of each focus group were made to be at least three participants per focus group as recommended by a guideline [47]. Some demographic characteristics of the participants in respective focus groups were listed in Table 1.

Thematic analysis of six FGDs identified six major particulars that should be known by patients prior to SDM implementation. The information will be beneficial for developing a strategic tool to promote SDM in the management of depression. Patient is the best person to provide the information. Therefore, from the FGDs sessions with patients, several data had been obtained, in order to be included in the prospectus SDM guideline. Additionally, perspective of doctors who are attending this kind or patients were also taken into consideration from respective FGDs sessions with doctors, so that the information obtained was clarified to be suitable with current practice.

### 3.1. Theme 1: Summary of Treatment Options

Patients stated that they would like to know the options of treatment available for their current conditions. They believed that doctors have all the information related to the advantages and disadvantages of each treatment option. The only patients needing layman explanations was the highlight of the important points that they should know, before initiating the treatment plan.

“I think if he is able to give a summary of each of the options, then highlight the pros and cons.”

(P009, FGD 2, Patient)

When patients came to see their doctors, they were expecting that doctors will give some words that show support to the patients’ illness. By giving a summary of treatment options available, the doctors can give an overview of the types of medicine that the patient will get after this. Then, the patients’ condition can be improved as a result of understanding the importance and role of each medicine.

“Since I came here, I need the medicine, to improve my condition or others, I need treatment from medicine. Maybe some counselling, maybe some support from the doctor.”

(P008, FGD 2, Patient)

From the doctors’ side, they agreed that a summary of treatment options available should be given to the patients before any decision of specific treatment is made. Currently, patients were directly instructed to follow any decision made by doctors, with information or feedback seldom being obtained first from patients.

“Maybe what we are lacking now is that we never ask their information, what do you know about treatment, if you think your treatment is helpful, how your treatment will be helpful. If your treatment is not helpful, what you…. We never try to gain information from them. We always give our words.”

(P002, FGD 1, Doctor)

### 3.2. Theme 2: Correct Ways of Taking Medication

Sometimes patients confused about the proper administration of the medications prescribed to them. Some medications may have different ways of administration. As an example, Remeron—or its generic name, Mirtazapine—is an orally disintegrating tablet. Patients should be instructed to make sure their hands are dry when opening the tablet blister pack. Then, the tablet should be placed immediately on the tongue. Once the tablet is removed from its blister, it cannot be stored. Should there be any discrepancies, the patients should take proper action that they think is appropriate.

“I had a bad experience last time. She gave me the medicine. She gave me Remeron. You know you have to put it under your tongue, right? And then my tongue got swollen. You know, it’s night time. And I have to call her next day. Luckily I have her number.”

(P018, FGD 2, Patient)

On top of that, doctors concerned about the understanding of patients related to information of treatment available that will be presented. The information should be summarized in the simplest language that the patients can understand, so that they can know the relationship of this treatment option with their diseases. This can also be considered as giving health literacy to the patients to further improve their understanding of their treatment journeys.

“I think without any health literacy, there will be no SDM at all. They need around a basic level of health literacy or understanding of their disease.”

(P006, FGD 1, Doctor)

### 3.3. Theme 3. Potential Side Effects of Treatments Related to Patients

Patients preferred to have an overview of potential side effects that they probably get from the medications prescribed. Usually, when doctors communicate with patients, they will find out that side effects are a frequent concern. Patients need to know this point earlier so that precautionary measures can be taken into consideration to avoid other adverse reactions.

“Because, when you communicate with the patient, you can find out a lot of things. Like what you said, what are the side effect of the medication, whether it is suitable for the patient or not, if the doctor just prescribed the medicine then there might the wrong medications for the patient. So, it’s good to practice SDM.”

(P007, FGD 2, Patient)

However, if side effect information is going to be presented after the patients and doctors communicate a lot of thing together, more consultation will be needed. Even under current practice, both patients and doctors complained about long waiting times as a result of the long consultation process.

“It’s just really taking some time to give some information for the patient or family to choose something, which is required anyways.”

(P009, FGD 2, Patient)

Particularly, outpatient clinics may have peak hour periods that present a lot of patients to be attended to in a very short time. This may limit the presentation of information of side effects, if it is not prioritized in advance of other conversations.

“You got a busy clinic, a lot of patients, you might not spend a lot of time in clinic, explaining treatment to the patients.”

(P011, FGD 3, Doctor)

### 3.4. Theme 4: Sharing of Case Study Related to Treatment Options

Patients really appreciate if their doctors can give examples of previous patients’ experiences that were related with their disease and treatment. People tend to believe the situation that had happened close to them. The similarity of characteristics related to the individual involved will influence the patients’ response towards their medications after this.

“Maybe the doctor could share with us some examples of cases that are very similar to our own, so we have some examples of the side effect or the negative things especially, coming from the people, samples that are very close to us. And then there will be a better judgment. That means in another word, more sharing of his expertise, not just on the medicine but the impact on the clients.”

(P009, FGD 2, Patient)

### 3.5. Theme 5: Cost of Treatment Options

Even though patients in the FGD sessions did not mention any issues about treatment costs, some doctors in the FGD sessions stated they have encountered patients who were really concerned about treatment costs. Doctors were commonly explaining about this issue and providing solutions to the needy patients. Sometimes, the welfare department may be helpful in assisting patients to buy particular medications.

“The issues would be, how much the cost of medication?”

(P022, FGD 5, Doctor)

Some doctors mentioned that this issue had already been taken into consideration. Alternative therapy was also provided as a solution, based on patients’ preferences.

“And for the issues of patient concern, I think all of them have mentioned; dependence, side effects, costs. And some of them don’t want to be on medication, probably psychotherapy, and talk therapy. And alternative therapy is always there—no matter Chinese, Malay, Indian—in KL.”

(P027, FGD 5, Doctor)

### 3.6. Theme 6: Input from Pharmacist

Depression is a disease that should be managed collaboratively. Other health care professionals were mentioned by patients and doctors. The most commonly stated profession to be involved is the pharmacist.

“Because, I think the doctor would already have his own agenda of what he needs to say, and maybe during the time when we get the medicine, we can have more interaction with the pharmacist.”

(P009, FGD 2, Patient)

“If there’s an issue about the medication, pharmacists can also help us, to counsel them, like insulin, how many mg do you get, how to inject it.”

(P003, FGD 1, Doctor)

However, the doctors mentioned that they hardly to see involvement of pharmacists in current psychiatric service. They can only remember interaction with pharmacists in other departments. Obviously, this issue needs to be taken into serious consideration.

“I think in Pediatrics, when I was doing my housemanship. Usually the pharmacist, they will follow the rounds, they will give suggestions, because there are alternatives to medication, can give inputs la, which one has less side effects. So, they are involved.”

(P014, FGD 3, Doctor)

“Yes, but not psychiatry department. Most of the time other departments, medical, peds. My experience is that the pharmacist will follow the rounds, and they will only answer the doctor’s doubts without giving much suggestion. For example, when the consultant has doubts on drugs, the interaction, then they ask the pharmacist. But other than that, normally they don’t interfere in the clinical decision.”

(P015, FGD 3, Doctor)

## 4. Discussion

This study, to our knowledge, is the first qualitative study in Malaysia aiming to develop strategic tool for antidepressant use among patient with major depressive disorder. The findings of this study suggest that patients and doctors are aware of the importance of SDM in the management of depression. They provided useful information to be included in the development of a strategic tool to promote and implement SDM in depression management in Malaysia. Among the important information to be included is a summary of treatment options, correct ways of taking medication, potential side effects of treatments related to patients, sharing of case study related to the treatment options, cost of treatment options, and input from pharmacist. These six points may be further elaborated so that a specific checklist for this tool can be developed. 

Firstly, the summary of treatment options may include brand name, generic name, indications, and drug classifications. Secondly, the correct ways of taking medication may include detail of medication dosing, route of administration and monitoring parameter that should be done by doctor and patient after this. Thirdly, potential side effects of treatments related to patients may include adverse effects, black box warning, contraindication, and possible drug–drug interactions with the patients. All the information stated can easily be obtained from a subscribed database from DynaMed (www.dynamed.com).

Fourthly, sharing of case study related to the treatment options may include any simple case report from the doctors’ own experiences. The next point is the cost of treatment options, which may list out simple price comparison of medications. Even though the cost aspect was obtained from FGD with doctors, it was the common things requested by their patients during clinic consultation. Lastly, input from pharmacist may include the explanation of simple mechanisms of action/pharmacokinetics of the selected drugs and double check of medication prescribed for patient safety. The summary of these points can be seen in Table 2.

The most important thing we found from this study is that patients are not keen to know efficacy of the antidepressants based on scientific literatures. Even though doctors will only prescribe evidence-based medicine (EBM) to their patients, this point is not becoming a crucial fact for patients to know. The patients only interested to know the effectiveness of the prescribed medicine based on examples of cases attended by doctors, previously. This is known as patient-centered medicine (PCM), which is another part of movements in health system. PCM has a personalized approach, that is focusing on individual health outcomes improvement, whereas EBM has a generalized approach, that is preferring clinical trials to obtain the best result for the average patient [48].

The information from this study can be used to create an evidence-based tool to support SDM in the use of antidepressants among patients with major depressive disorder. Doctors can access and use this tool to engage with the depressed patients in order to achieve the intended management outcomes. Our findings are in line with recent systematic review of tools to promote SDM in serious illness [49] and fulfil the need for adoption of SDM in social and psychiatric services [35,50,51]. Therefore, this strategic tool will be further tested in real psychiatric clinic sessions after this.

From our findings, we found that our setting has fulfilled the three basic prerequisites of SDM implementation in care settings [1]: (1) attending health care providers are willing and have the ability to include patients in decision-making activities; (2) patients are willing and have the ability to participate actively in the decision-making activities; and (3) additional decision support and information are available to assist the process of SDM [52,53,54]. The decisional and information needs will include the information related to mental health services for patients as well as the process of acquiring necessary knowledge and information about patients’ goals, life situations, and experiences. We can adapt a three-step model for clinical practice to promote SDM as developed by Elwyn et al. [55]. This model consists of several steps of “choice talk, option talk, and decision talk” which will be applied to support the development of this strategic tool for shared decision-making in the use of antidepressants among patients with major depressive disorder.

The results from a recent study in mental health care user [1] proved that the model by Elwyn et al. [55] which had been used in somatic care related SDM previously, was also suitable for mental health services, in terms of content and process. However, two additional steps were suggested to improve the current model. The initial step should begin with a preparation phase, which involves development and description of issues related to patients and health care providers. The last steps should be ended by a follow-up phase, which include identification of needs for further contacts between patients and health care providers after a decision has been made. All steps of this model can be seen in Table 3.

Therefore, information from Table 2 can be put into the model shown in Table 3. All these contents and processes can be delivered to the patients during the outpatient clinic session, as shown by a good example of a web-based application, which is known as “Common Ground”, in order to support recovery and SDM in psychiatric medication clinics [56]. In brief, this web-based intervention involved the waiting time of patients prior to see doctors. A peer staff member will approach the patient who is waiting for their turn to see doctor. The patient will be guided by the peer staff to use the computer, or any gadget provided by the clinic, in order to use this website. On the website, there are several questionnaires to be answered by the patient. We can include relevant questions based on information and process that have been identified in this study. Eventually, all the results of the questionnaires will be presented to the attending doctors, and SDM process will be continued with the patients, based on information obtained. Besides the web-based intervention, interactive antidepressant guidelines were implemented on smartphone applications to allow patients to search for information when they are “on the go” and outside the clinical settings [57]. As patients with depression were more engaged with social media [58] and smartphones [59], the “Common Ground” application can be modified to incorporate the latest information technology to reach more patients.

Nevertheless, there are some limitations of this study. Perception and views of those represented in our study may not be transferable to all patients and doctors in Malaysia. It may be subjected to social desirability bias [60]. Less junior doctors or patients may have been unwilling to challenge the beliefs of senior colleagues. To address this potential problem, junior doctors or younger patients were arranged to sit facing the moderator during each focus group session. This permitted more direct engagement with them and was assisted by giving direct questions for clarification and agreement. However, the “member checking” was not completely done, in order to limit the potential lengthy session taken for finishing the discussion flow. Additionally, coding of transcribed data is an interpretive and subjective experience. There are possibilities that the researcher may not interpret or identify inference data precisely. However, themes were derived as data saturation was achieved and researchers had previous training in qualitative research and checking of coding by the research team.

## 5. Conclusions

The perspectives of doctors and patients regarding SDM in the use of antidepressants among patients with major depressive disorder have been discussed in depth. A strategic tool has been developed to inform a transition from a traditional ‘paternalistic’ model of clinical decision making into a current ‘informed’ decision making model in local situation. Patients’, doctors’ and pharmacists’ roles on the promotion and implementation of this tool have been highlighted. The proposed tool can be used to inform further research efforts and developments that move beyond identifying barriers to developments that contribute towards the successful and appropriate implementation of SDM into clinical practice.

## Figures and Tables

**Table 1:** Selected demographic characteristics (*n* = 30) of each focus group discussion (FGD).

Variables	FGD 1	FGD 2	FGD 3	FGD 4	FGD 5	FGD 6
Date *	20 Sept. 2017	21 Sept. 2017	27 Sept. 2017	28 Sept. 2017	4 Oct. 2017	12 Oct. 2017
No. of participants	6	4	7	4	6	3
Gender	2 M, 4 F	1 M, 3 F	5 M, 2 F	4 F	4 M, 2 F	3 F
Role in depression management	Doctor	Patient	Doctor	Patient	Doctor	Patient

* Date of conducting the FGD session; M = male, F = female.

**Table 2:** Selected information to be included into the strategic tool.

No.	Information Needed for SDM	Description
1	Summary of the treatment options	Brand name, generic name, indications, and drug classifications
2	Correct ways of taking medication	Medication dosing, route of administration and monitoring parameter
3	Potential side effects of treatments	Adverse effects, black box warning, contraindication and possible drug–drug interactions
4	Sharing of case study related to the treatment options	Any simple case report from the doctors’ own experiences
5	Cost of treatment options	Simple price comparison of medications
6	Input from pharmacist	Simple mechanism of action/pharmacokinetics of the selected drugs and double check of medication prescribed for patient safety

**Table 3:** A model for SDM as adapted from Grim, Rosenberg, Svedberg, & Schön, 2016.

1. Preparation	2. Choice Talk	3. Option Talk	4. Decision Talk	5. Follow-Up
Develop agenda, provide patient decision support	Step back, offer choice, justify choice-preferences matter, check reaction, defer closure	Check knowledge, list options, describe options-explore preferences, harms and benefits, summarize	Focus on preferences, elicit preferences, move to a decision, offer review	Accessible contact, planned follow-up, possibility to reconsider

## Supplementary Materials

The following are available online at http://www.mdpi.com/1660-4601/15/7/1402/s1, “Focus Group Topic Guide” which provides the focus group discussion guide for this study.
