# Supplementary material for: Development of a Strategic Tool for Shared Decision-Making in the Use of Antidepressants among Patients with Major Depressive Disorder: A Focus Group Study
Source: Int J Environ Res Public Health. 2018 Jul 3;15(7):1402. doi: 10.3390/ijerph15071402 (PMC6068982; doi:10.3390/ijerph15071402)
Supplement: Supplementary file 1 [file ijerph-15-01402-s001.pdf]

Supplementary Materials: Development of a strategic tool for shared decision-making in the use of antidepressants among patients with major depressive disorder: A focus group study

Syahrir Zaini, Harvin Anbu Manivanna Bharathy, Ahmad Hatim Sulaiman, Jesjeet Singh Gill, Koh Ong Hui, Hasniza Zaman Huri, Siti Hadijah Shamsudin and Ng Chong Guan

1. Topic Guide—Semi-Structured

1.1. Opening

Tell us, have you ever heard about shared decision-making (SDM), before?

1.2. Introductory

What are your thoughts about this SDM?

1.3. Transition

How do you feel about this SDM as compared with current psychiatric clinic service, that you have experienced, currently?
How do you think this SDM can prevent relapse in patients with mood disorder?

1.4. Key Questions

How patients and psychiatrists can negotiate what kind of treatment is chosen?
What can patients contribute so that we reach decisions that are reasonable for both patients and psychiatrists?
What else can patients do to contribute to successful treatment?

1.5. Ending Questions

If you had a chance to give advice to the director of this service, what advice would you give?
Let's recap our discussion for today. Our aim is get your opinion in evaluating this new service. We want to know how to improve the service.

1.6. Concluding

Briefly, this is the summary of our discussion. The main points that I can extract are …
Do you have any comments, amendments or corrections to this summary?

1.7. Final question

Have we missed anything?
Do you think we've missed anything in the discussion?
This is the first in a series of groups like this that we are doing. Do you have any advice for how we can improve?

© 2018 by the authors; licensee MDPI, Basel, Switzerland. This article is an open access article distributed under the terms and conditions of the Creative Commons by Attribution (CC-BY) license (http://creativecommons.org/licenses/by/4.0/).
